# Analyzing and evaluating the metabolic and endocrine characteristics between lean and obese patients with polycystic ovary syndrome: a systemic review and meta-analysis

**DOI:** 10.3389/fendo.2025.1680685

**Published:** 2025-10-14

**Authors:** Caiyu Zheng, Yuhao Lin, Zhijun Zhang, Jiawen Ye, Yanmei Lin, Jianqing Tian

**Affiliations:** ^1^ Fujian Medical University Xiamen Humanity Hospital, Xiamen, Fujian, China; ^2^ School of Medicine, Xiamen University, Xiamen, Fujian, China; ^3^ The School of Clinical Medicine, Fujian Medical University, Fuzhou, Fujian, China

**Keywords:** lean PCOS, obese PCOS, metabolic, endocrine, meta-analysis

## Abstract

**Objective:**

We performed an extensive meta-analysis to compare hormone levels and metabolic attributes between obese PCOS (OP) and lean PCOS. The main outcome of the study was the differences in critical clinical parameters, including luteinizing hormone (LH), follicle-stimulating hormone (FSH), low-density lipoprotein (LDL) cholesterol levels, systolic and diastolic blood pressures (BPs), and fasting blood sugar (FBS) levels between lean and OP patients.

**Methods:**

The present systematic review and meta-analysis was conducted in line with the PRISMA (Preferred Reporting Items for Systematic Reviews and Meta-Analyses) guidelines and the protocol of this study was prospectively registered on PROSPERO (Registration No. CRD420251039530) to minimize reporting bias and enhance transparency. Briefly, a comprehensive search was performed on the PubMed, ISI Web of Science, Embase and Cochrane Central Register of Controlled Trials from inception to Apr. 1, 2025, in any language, with the exclusion of abstract-only publications.

**Results:**

Seventy-three studies were analyzed. There were marked differences in metabolic indicators between the two groups. Lean PCOS participants had slightly lower levels of diastolic (SMD −0.56, 95% CI −0.79 to −0.33, p < 0.01) and systolic (SMD −0.58, 95% CI −0.80 to −0.36, p < 0.0003) BP relative to individuals with obese PCOS. They had lower levels of LDL (SMD −0.49, 95% CI −0.60 to −0.38, p < 0.01) and triglycerides (SMD −0.72, 95% CI −0.85 to −0.59, p < 0.01) than obese PCOS participants. The LH/FSH ratio in lean PCOS patients exceeded that in obese PCOS patients (SMD 0.23, 95% CI 0.07 to 0.40, p < 0.01). Moreover, the homeostasis model assessment of insulin resistance (HOMA-IR) was higher in obese PCOS patients (SMD -0.88, 95% CI -1.03 to -0.72, p < 0.01). However, there were no significant differences in anti-Müllerian hormone (AMH) level between the two groups.

**Conclusion:**

This meta-analysis provides valuable information regarding the profile of metabolic and endocrine characteristics between lean and obese PCOS patients. The specific treatment approach should be customized to each patient’s symptoms, fertility needs, and overall health. Further research is advocated to investigate the underlying mechanisms and to develop more targeted treatment strategies for different subgroups of PCOS patients.

## 1 Introduction

Polycystic ovary syndrome (PCOS) is the most prevalent cause of ovulatory infertility, affecting about 90–95% of women with anovulatory infertility (1). Studies have shown that PCOS is associated with long-term metabolic complications such as T2DM and dyslipidemia, and it affects approximately 10% of women of reproductive age (2).

Clinically, PCOS is characterized into two phenotypes, overweight/obese and lean, the latter being a much less common presentation of the syndrome. Obesity is a major risk factor of PCOS, underscoring the need to develop obesity treatments for women with PCOS (3). A previous study found that a 1% increase in obesity elevated the risk of PCOS by 0.4% based on the Rotterdam criteria (4). Insulin resistance (IR) in PCOS may develop independently of obesity but may also be exacerbated by obesity, with studies estimating a prevalence of 75% in lean and 95% in overweight PCOS women (5). In a meta-analysis, metabolic disorder of polycystic ovary syndrome in adolescents was worsened by obesity. Specifically, obese PCOS (cases group) had significantly lower sex hormone-binding globulin (SHBG) and High-Density Lipoprotein Cholesterol (HDLC) levels, but the levels of triglyceride, leptin, fasting insulin, low density lipoprotein cholesterol and free testosterone levels were significantly higher with normal weight PCOS adolescents (6).

However, in 100 women with PCOS, insulin resistance was associated with PCOS, irrespective of whether the subject was lean or obese, clinical and hormonal profile was similar to PCOS patients with elevated BMI (7). A systemic review found no differences in clinical manifestation of PCOS between the lean and overweight subgroups, such as hirsutism and hyperandrogenism (8).

Considering the above studies, we postulate that there are differences in the specific metabolic disorders associated with lean polycystic and obese polycystic. In this study, we performed an extensive meta-analysis to compare hormone levels and metabolic attributes between obese PCOS and lean PCOS.

## 2 Data sources and search strategy

The present systematic review and meta-analysis was conducted in line with the PRISMA (Preferred Reporting Items for Systematic Reviews and Meta-Analyses) guidelines and the protocol of this study was prospectively registered on PROSPERO (Registration No. CRD420251039530) to minimize reporting bias and enhance transparency (9). Briefly, a comprehensive search was performed on the PubMed, ISI Web of Science, Embase and Cochrane Central Register of Controlled Trials (CENTRAL) from inception to Apr. 1, 2025, in any language, with the exclusion of abstract-only publications. The search strategy was designed by an experienced librarian using the Medical Subject Headings and relevant key words which comprised of the “Polycystic Ovary Syndrome”. The complete search strategy is presented in the online **Supplementary Table 1**. Other relevant publications and unpublished trials were identified through a manual search.

### 2.1 Inclusion criteria

The following selection criteria were adopted: (a) studies based on animal models were excluded. (b) lean was defined as individuals with BMI ≤ 25 kg/m2, and for Asian populations, BMI ≤ 23 kg/m^2^, (c) overweight/obese individuals were those with a BMI of ≥25.0 kg/m^2^, and for the Asian population, a BMI ≥23 kg/m^2^, (d) studies comparing lean PCOS (LP) with overweight/obese PCOS.

### 2.2 Data extraction and quality assessment

Studies that did not meet the pre-set eligibility criteria were excluded during the preliminary screening phase of relevant studies. Duplicated studies were excluded using the EndNote reference library program. The selected articles were further reviewed to identify the existence of different versions of the articles and those with accessible full texts. To enhance consistency and accuracy in the extracted data, four authors were involved in data extraction, which included all outcomes of interest and baseline characteristics. The main outcome was differences in metabolic and hormonal indicators between the lean PCOS and obese PCOS individuals. Moreover, we investigated the development of systemic diseases and the prevalence of metabolic syndrome (MS) in lean versus obese PCOS (OP) patients. The secondary outcome of the study was the differences in critical clinical parameters, including low-density lipoprotein (LDL) cholesterol levels, triglycerides, systolic and diastolic blood pressures (BPs), and fasting blood sugar (FBS) levels between lean and OP patients. To enhance the integrity of the extracted data, any uncertainties arising during the data extraction process were addressed through collaborative discussions among the investigators involved in data extraction.

### 2.3 Statistical analysis

All statistical comparisons between lean PCOS and obese PCOS were performed using the Revman software (Version 5.4.1). Standard mean difference (SMD) and their respective 95% confidence intervals (CIs) were calculated for the two groups and the I^2^ statistic was employed, with values less than 50% indicating mild heterogeneity. In cases where there was significant heterogeneity, sensitivity analyses were conducted using the leave-one-out method. A p value below 0.05 indicated significant difference between the groups.

## 3 Results

### 3.1 Selection of studies

**Figure 1:**
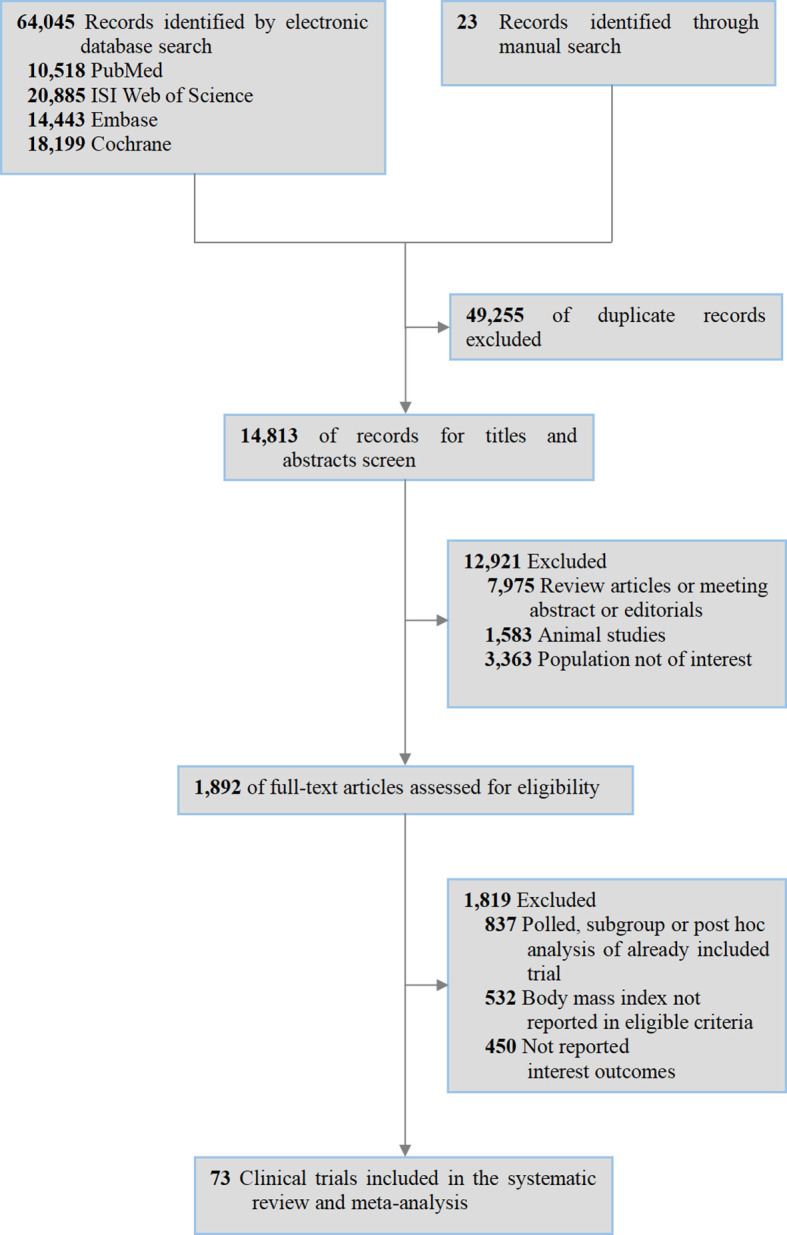
Flowchart.

Relevant studies were selected following the PRISMA 2020 guidelines. A total of 64,045 records were identified from the electronic databases (10,518 from PubMed; 20,885 from ISI Web of Science; 14,443 from Embase; 18,199 from Cochrane Library) and 23 via manual search. After excluding 49,255 duplicates, 14,813 records were further screened by reading the titles/abstracts, leading to the exclusion of 12,921 articles (7,975 as reviews/abstracts/editorials; 1,583 as animal studies; 3,363 involving irrelevant populations). Subsequently, the remaining 1,892 full-text articles were analyzed, among which 1,819 were excluded (837 as pooled/subgroup/*post-hoc* analyses of included trials; 532 without BMI in eligibility criteria; 450 lacking reported outcomes of interest). Finally, 73 clinical trials were included in the systematic review and meta-analysis (**Figure 1**).

### 3.2 Characteristics of the included studies

The 73 studies were from 18 countries grouped as follows: Turkey (n=14), China (n=12), India (n=11), and Italy (n=5). The sample sizes ranged from 22 to 458, with all studies stratifying participants into lean PCOS and obese PCOS groups. Lean PCOS counts per study ranged 10–352, obese PCOS 10–352. Regarding the age distribution of the participants (61 studies reported age) with those of lean PCOS group having the mean age of 20.25 (1.45)–34.4 (4.8) years; obese PCOS with a mean age of 20.43 (1.53)–34.7 (4.4) years (12 studies NA). Analysis of the BMI data (59 studies) revealed that the mean BMI of the lean PCOS group was 19.92 (3.51)–23.23 (1.65) kg/m², while that of obese PCOS was 26.36 (1.86)–36.8 (4.8) kg/m² (14 studies NA). Age and BMI were reported as mean (SD), mean (min-max), mean (SE), median (5-95% CI), mean (5-95% CI), or medians (interquartile ranges) (**Supplementary Table 2**).

### 3.3 Quality assessment

The methodological quality of the included studies was determined using the Newcastle-Ottawa Scale, covering Selection, Comparability, and Outcome domains; the results are shown in **Supplementary Table 3**. Most studies exhibited a minimal risk of bias, indicating they were highly reliable.

### 3.4 Fasting blood sugar and blood pressure

**Figure 2:**
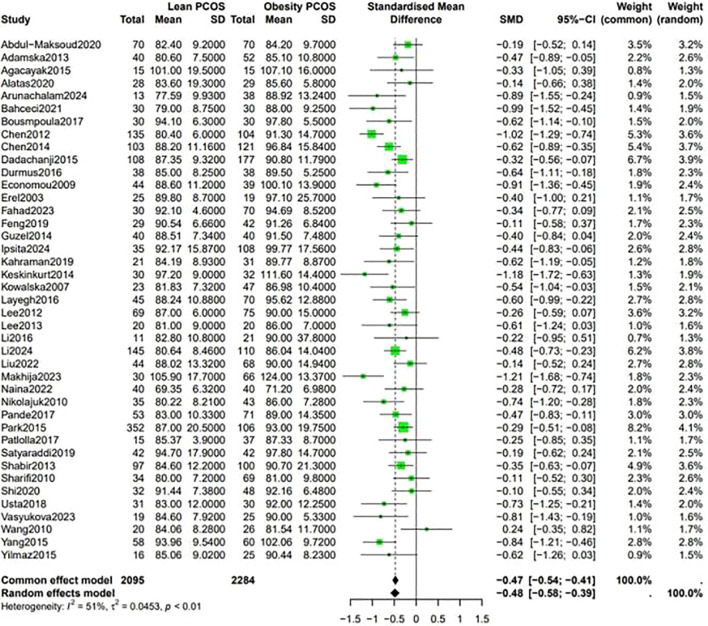
Forest plot for lean PCOS versus obese PCOS for FPG.

**Figure 3:**
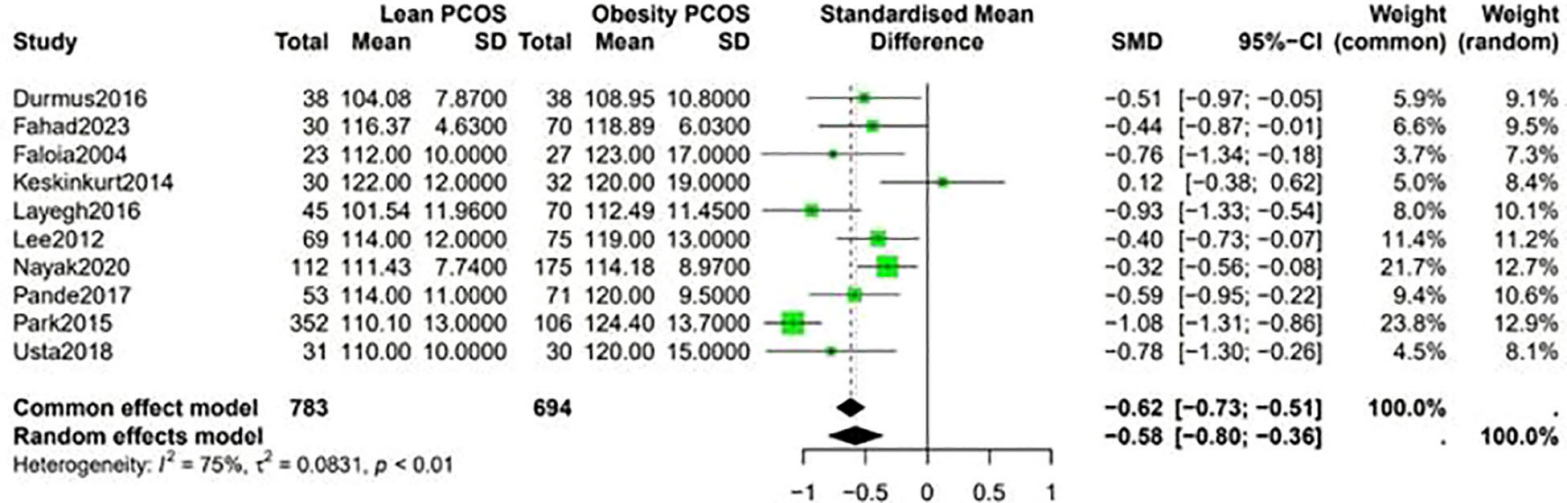
Forest plot for lean PCOS versus obese PCOS for BP.

Fasting blood sugar levels were lower in the lean PCOS compared with levels in the obese PCOS group (SMD −0.48, 95% CI −0.58 to –0.39, p <0.01) (**Figure 2**). Considering the BP measurements, lean PCOS participants had slightly lower levels of diastolic (SMD −0.56, 95% CI −0.79 to −0.33, p < 0.01) and systolic (SMD −0.58, 95% CI −0.80 to −0.36, p < 0.0003) BP relative to individuals with obese PCOS (**Figure 3**).

### 3.5 Lipid profile

**Figure 4:**
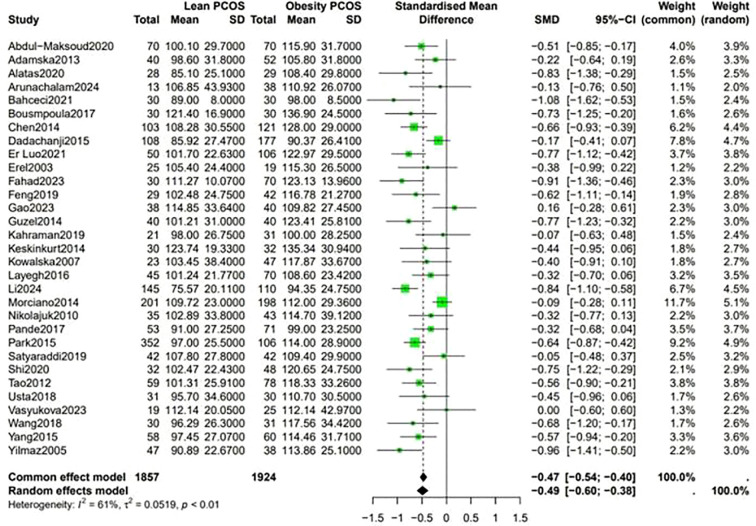
Forest plot for lean PCOS versus obese PCOS for lipid profile.

Analysis of the lipid profiles revealed that lean PCOS individuals presented with favorable outcomes compared with their obese PCOS patients. They had lower levels of LDL (SMD −0.49, 95% CI −0.60 to −0.38, p < 0.01) and triglycerides (SMD −0.72, 95% CI −0.85 to −0.59, p < 0.01). These differences in lipid levels indicate that lean PCOS may have a lower cardiovascular risk than obese PCOS (**Figure 4**).

### 3.6 Analysis of other endocrine characteristics

Comparative analysis demonstrated that endocrine hormone levels were significantly different between lean PCOS and obese PCOS patients.

**Figure 5:**
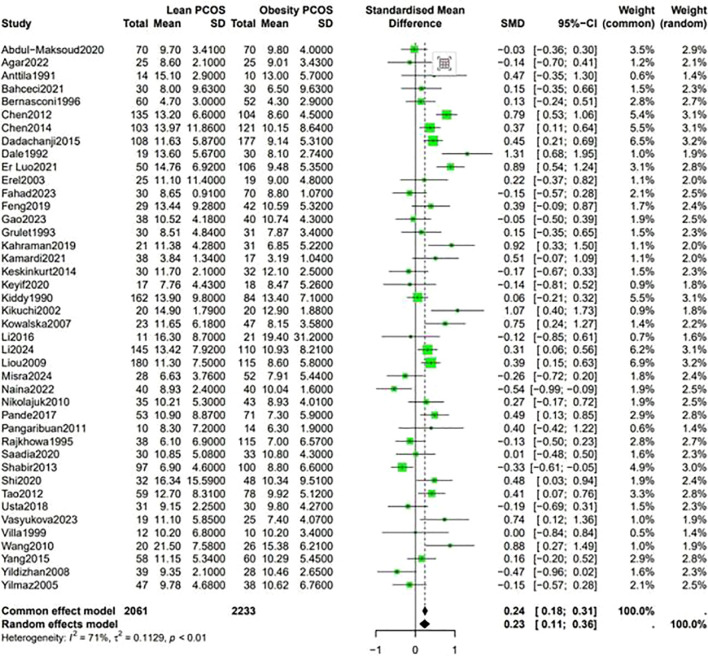
Forest plot for lean PCOS versus obese PCOS for LH.

**Figure 6:**
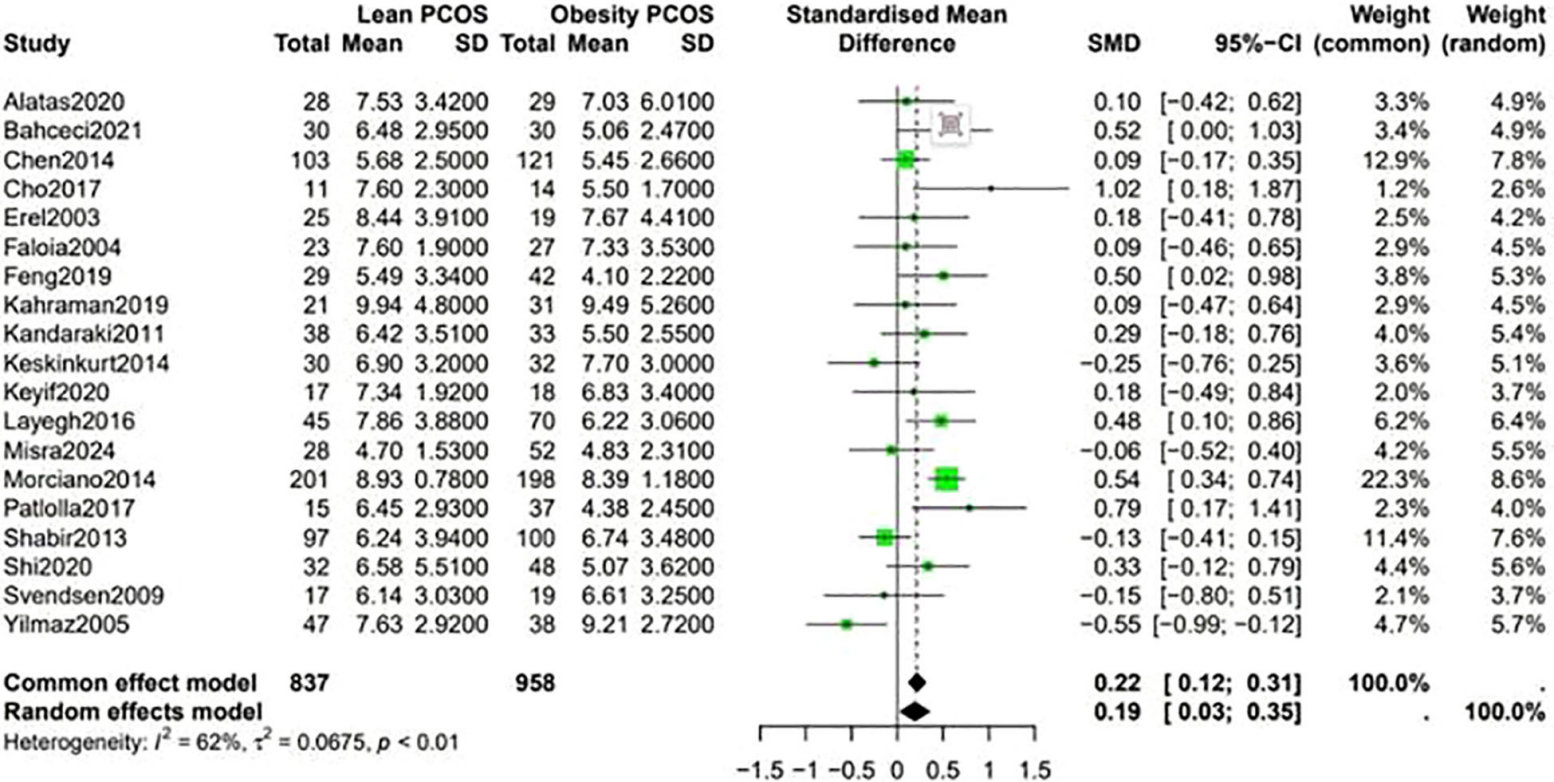
Forest plot for lean PCOS versus obese PCOS for DHEA-S.

Data shown in **Figure 5** indicate that the level of LH was higher in lean PCOS patients than in obese PCOS patients (SMD 0.23, 95% CI 0.11 to 0.36, p < 0.01). The level of FSH in lean PCOS patients was also higher than that in obese ones (SMD 0.10, 95% CI 0.04 to 0.17, p = 0.02). Moreover, the LH/FSH ratio in lean PCOS patients exceeded that in obese PCOS patients (SMD 0.23, 95% CI 0.07 to 0.40, p < 0.01). Notably, lean PCOS exhibited higher levels of SHBG compared to obese PCOS(SMD 0.81, 95% CI 0.61 to 1.02, p < 0.01). The levels of DHEA-S were significantly higher in the lean PCOS than in the obese PCOS patients(SMD 0.19, 95% CI 0.03 to 0.35, p < 0.01) (**Figure 6**).

**Figure 7:**
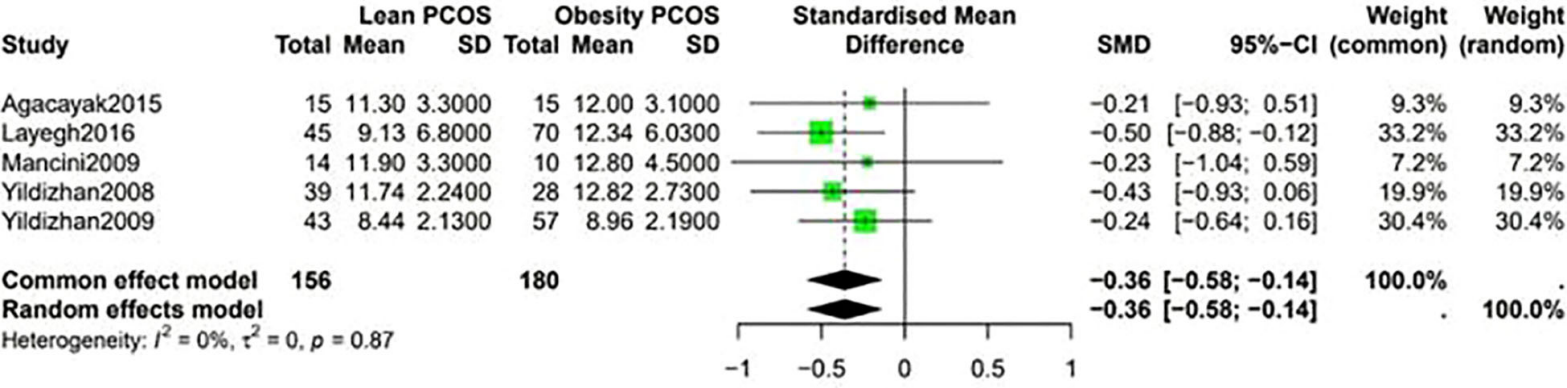
Forest plot for lean PCOS versus obese PCOS for Ferriman-Gallwey (F-G) score.

**Figure 8:**
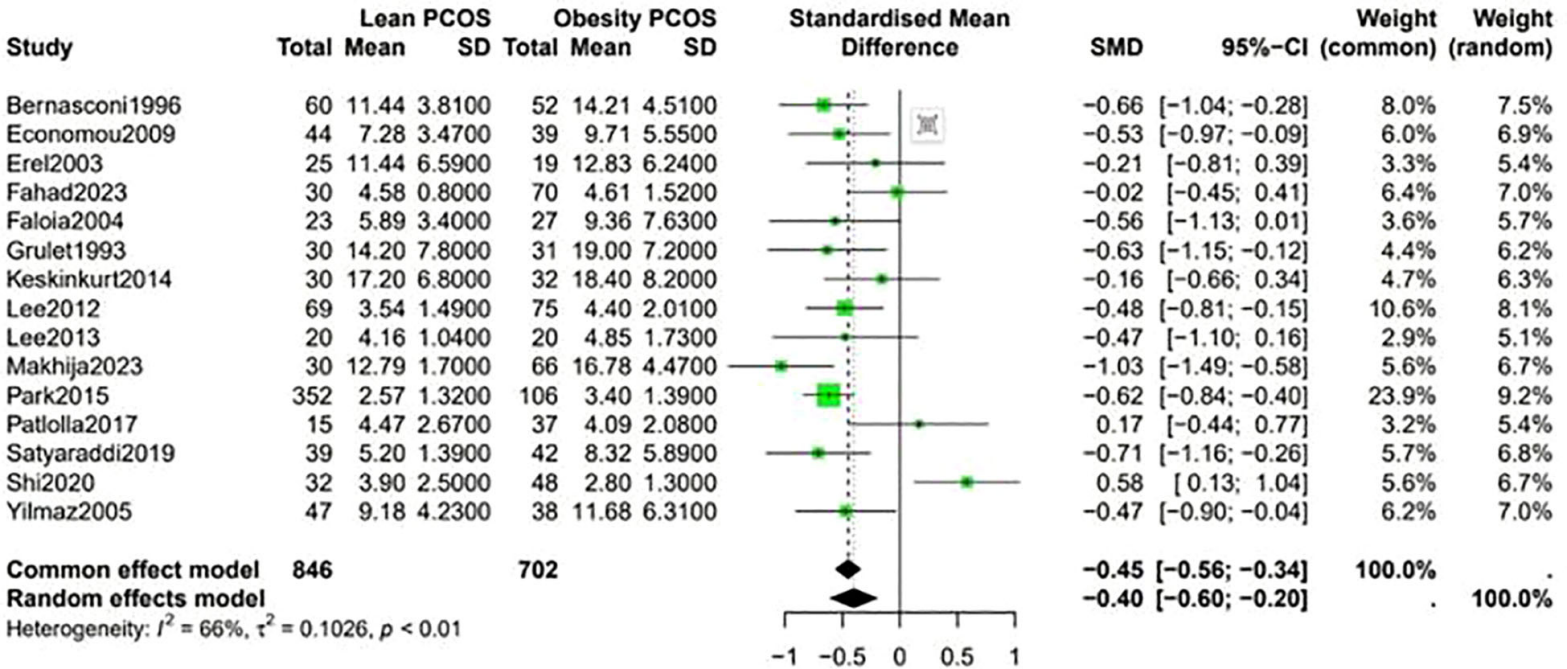
Forest plot for lean PCOS versus obese PCOS for F-T.

**Figure 9:**
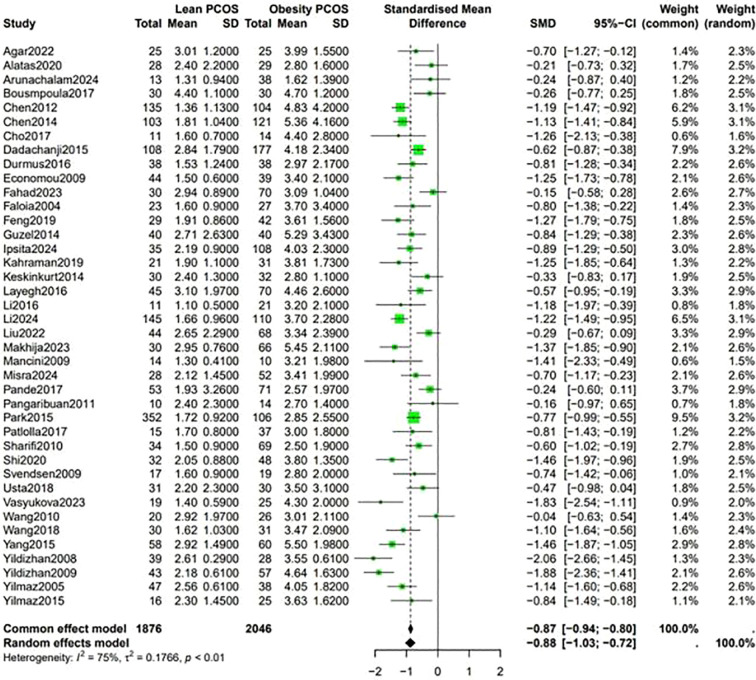
Forest plot for lean PCOS versus obese PCOS for (HOMA-IR).

A higher Ferriman-Gallwey (F-G) score was observed in obese PCOS individuals compared to lean PCOS patients (SMD -0.36, 95% CI -0.58 to -0.14, p = 0.87) (**Figure 7**). The levels of F-T were significantly different between the two groups, being higher in obese PCOS patients than in lean PCOS patients (SMD -0.4, 95% CI -0.6 to -0.2, p < 0.01) (**Figure 8**). Moreover, the homeostasis model assessment of insulin resistance (HOMA-IR) was higher in obese PCOS patients (SMD -0.88, 95% CI -1.03 to -0.72, p < 0.01), and their ovarian volume was also larger compared with that in the lean PCOS patients (SMD -0.17, 95% CI -0.32 to -0.01, p = 0.23) (**Figure 9**).

The analysis showed that there was no significant difference in AMH levels and androstenedione between the two groups.

## 4 Discussion

Here, we aimed to comprehensively analyze and compared differences between lean and obese PCOS patients. In contrast to previous studies, the present analysis performed a comprehensive examination of two major differences: metabolic characteristics and endocrine hormone levels between lean and obese PCOS patients. The findings of this study are expected to guide clinicians to formulate and develop targeted treatments.

### 4.1 Variations in metabolic indicators

These findings of this study indicate that obese PCOS patients have higher BP, blood lipid, and blood glucose levels compared to their lean counterparts, which is consistent with earlier reports (10). Notably, insulin resistance is a key feature of PCOS, and obesity has been implicated in the pathogenesis of this condition (11). In obese individuals, the expansion of adipose tissue increases the secretion of various adipokines and inflammatory substances. These compounds can disrupt insulin signaling pathways, thereby decreasing insulin sensitivity (12). Therefore, the body produces more insulin to maintain normal blood glucose levels. The increased insulin production stimulates lipid synthesis and storage in the liver and adipose tissue, thereby elevating blood lipid levels (13).

Our meta-analysis shows that, compared with obese patients with Polycystic Ovary Syndrome (PCOS), lean PCOS patients often present with a favorable metabolic profile. However, studies also indicate that when compared to the general population, lean PCOS patients are at a higher risk of developing metabolic disorders. Even if their body weight is within the normal range, they may have underlying metabolic problems such as mild insulin resistance or abnormal lipid metabolism (14).

### 4.2 Differences in endocrine hormone levels

#### 4.2.1 LH, FSH, and LH/FSH ratio

The analysis revealed that lean PCOS patients had higher levels of LH, FSH, and a higher LH/FSH ratio compared to obese PCOS patients. This highlights that neuroendocrine disturbances may be the most important mechanism in lean PCOS patients. The pathophysiology of both phenotypes (obese and lean PCOS) may be different (15).The hypothalamic-pituitary-ovarian (HPO) axis is an important player in the regulation of the reproductive hormones in the context of PCOS (16). In lean PCOS, dysregulation of the HPO axis can stimulate the excessive secretion of gonadotropin-releasing hormone (GnRH) by the hypothalamus. This, in turn, stimulates the pituitary gland to release more LH and FSH (17). A higher LH/FSH ratio is a typical characteristic of PCOS, which impairs the normal follicular growth and ovulation (18). In lean PCOS patients, this abnormal ratio may be more pronounced, potentially inducing more severe ovulation problems (19). Currently, The first-line management of PCOS patients is to adopt a healthy lifestyle through exercise and diet. It does not seem to significantly improve patients with lean PCOS, especially in relation to ovulation and infertility (20).

#### 4.2.2 SHBG and DHEA-S

One of the most important finding of this study is that lean PCOS patients had higher levels of SHBG and DHEA-S. SHBG is a glycoprotein that binds to sex hormones, mainly testosterone and estradiol, in the blood (21). In lean PCOS, elevated SHBG levels can potentially reduce the availability of free androgens, thereby alleviating the symptoms of hyperandrogenism to some extent (22). DHEA-S is a precursor of adrenal androgens, and its higher levels in lean PCOS may indicate that adrenal androgen synthesis is involved in the pathophysiology of this subgroup (23). In lean PCOS, adrenal glands are overactivated, thereby increasing the production of DHEA-S. This can then be converted into more potent androgens in peripheral tissues.

#### 4.2.3 Androgens, F-G score, and F-T

Obese PCOS patients exhibited higher F-G scores and F-T levels. Studies have shown that obesity can exacerbate hyperandrogenism in PCOS via diverse mechanisms. Insulin resistance, which is more common in obese PCOS, can increase the activity of ovarian theca cells, thereby increasing androgen production (24). Moreover, adipose tissue can convert adrenal androgens into more potent forms, such as testosterone, through reactions catalyzed by enzymes such as, 5α-reductase (25). The higher F-G scores and F-T levels in obese PCOS patients reflect more severe hyperandrogenism, which is often associated with clinical symptoms such as hirsutism and acne (24).

#### 4.4.4 Ovarian volume and AMH

The larger ovarian volume in obese PCOS patients may be driven by an increase in the number of small antral follicles and thickening of the ovarian stroma, which are hallmark features of PCOS (26). In obese PCOS, excessive androgen production and abnormal hormonal environment promotes follicular recruitment and growth but also prevents the normal follicular maturation and ovulation. This induces the accumulation of small follicles in the ovaries and an increase in ovarian volume (27). The lack of significant differences in AMH levels between the two groups is consistent with findings from recent studies, suggesting that AMH may not be directly related to body weight in PCOS (28).

### 4.3 Clinical significance

The differences in metabolic and endocrine characteristics between lean and obese PCOS patients may have important implications for clinical practice. For obese PCOS patients, weight management should be incorporated in the treatment plan. Lifestyle modifications, such as a balanced diet and regular exercise, can potentially alleviate insulin sensitivity, reduce androgen levels, and restore ovulatory function (29). Moreover, it has been shown that metformin is more effective as an ovulation stimulation agent when administered to non-obese women with PCOS (30). For lean PCOS patients, although their metabolic status may be relatively better, they still require monitoring to track their metabolic parameters. In some cases, appropriate treatments for ovulatory dysfunction and hyperandrogenism should be administered. The specific treatment approach should be customized to each patient’s symptoms, fertility needs, and overall health.

### 4.4 Advantages and limitations

The results of this study are valuable in terms of several strengths. We utilized a comprehensive literature search, stringent inclusion and exclusion criteria to identify eligible studies. First, to the best of our knowledge, this is the first meta-analysis to evaluate the metabolic and endocrine characteristics between lean and obese patients with polycystic ovary syndrome (PCOS). Second, the present study comprehensively compared the metabolic and endocrine characteristics of lean and obese PCOS, providing important data to guide the clinical classification of patients. Moreover, it clarifies the differences in key indicators between the two types of patients, which is essential to the implementation of targeted treatment. However, the included studies exhibited some degree of heterogeneity, and differences in diagnostic criteria and detection methods, which may affect the stability of the results. In addition, there was selection bias since the samples were mainly selected from specialized outpatient clinics, implying lack of population representativeness.

In conclusion, this meta-analysis provides valuable information regarding the profile of metabolic and endocrine characteristics between lean and obese PCOS patients. Further research is advocated to investigate the underlying mechanisms and to develop more targeted treatment strategies for different subgroups of PCOS patients.

## References

[1] Sirmans S Pate K. Epidemiology, diagnosis, and management of polycystic ovary syndrome. Clin Epidemiol. (2013). doi: 10.2147/clep.S37559, PMID: 24379699 PMC3872139

[2] Floyd R Hughes N O’Sullivan L Hevey D Murphy N Hinds C. A prospective study of antenatal anxiety and depression in pregnant women with polycystic ovary syndrome. Irish J psychol Med. (2024), 1–5. doi: 10.1017/ipm.2024.56, PMID: 39581902

[3] Lim SS Davies MJ Norman RJ Moran LJ. Overweight, obesity and central obesity in women with polycystic ovary syndrome: a systematic review and meta-analysis. Hum Reprod Update. (2012) 18:618–37. doi: 10.1093/humupd/dms030, PMID: 22767467

[4] Amiri M Hatoum S Hopkins D Buyalos RP Ezeh U Pace LA. The association between obesity and polycystic ovary syndrome: an epidemiologic study of observational data. J Clin Endocrinol Metab. (2024) 109:2640–57. doi: 10.1210/clinem/dgae488, PMID: 39078989

[5] Joham AE Boyle JA Zoungas S Teede HJ. Hypertension in reproductive-aged women with polycystic ovary syndrome and association with obesity. Am J Hypertension. (2015) 28:847–51. doi: 10.1093/ajh/hpu251, PMID: 25542625

[6] Li L Feng Q Ye M He Y Yao A Shi K. Metabolic effect of obesity on polycystic ovary syndrome in adolescents: a meta-analysis. J Obstetrics Gynaecology. (2017) 37:1036–47. doi: 10.1080/01443615.2017.1318840, PMID: 28657375

[7] Prakash A Saxena P Nigam A Mishra A. Polycystic ovary syndrome: Is obesity a sine qua non? A clinical, hormonal, and metabolic assessment in relation to body mass index. Indian J Endocrinol Metab 16. (2012). doi: 10.4103/2230-8210.103011, PMID: 23226650 PMC3510975

[8] Toosy S Sodi R Pappachan JM. Lean polycystic ovary syndrome (PCOS): an evidence-based practical approach. J Diabetes Metab Disord. (2018) 17:277–85. doi: 10.1007/s40200-018-0371-5, PMID: 30918863 PMC6405408

[9] Page MJ McKenzie JE Bossuyt PM Boutron I Hoffmann TC Mulrow CD. The PRISMA 2020 statement: an updated guideline for reporting systematic reviews. Bmj. (2021). doi: 10.1136/bmj.n71, PMID: 33782057 PMC8005924

[10] Goodarzi MO Dumesic DA Chazenbalk G Azziz R. Polycystic ovary syndrome: etiology, pathogenesis and diagnosis. Nat Rev Endocrinol. (2011) 7:219–31. doi: 10.1038/nrendo.2010.217, PMID: 21263450

[11] Diamanti-Kandarakis E Dunaif A. Insulin resistance and the polycystic ovary syndrome revisited: an update on mechanisms and implications. Endocrine Rev. (2012) 33:981–1030. doi: 10.1210/er.2011-1034, PMID: 23065822 PMC5393155

[12] Fain JN Madan AK Hiler ML Cheema P Bahouth SW. Comparison of the release of adipokines by adipose tissue, adipose tissue matrix, and adipocytes from visceral and subcutaneous abdominal adipose tissues of obese humans. Endocrinology. (2004) 145:2273–82. doi: 10.1210/en.2003-1336, PMID: 14726444

[13] Grundy SM. Metabolic syndrome pandemic, arteriosclerosis. Thrombosis Vasc Biol. (2008) 28:629–36. doi: 10.1161/atvbaha.107.151092, PMID: 18174459

[14] Deligeoroglou E Vrachnis N Athanasopoulos N Iliodromiti Z Sifakis S Iliodromiti S. Mediators of chronic inflammation in polycystic ovarian syndrome. Gynecological Endocrinol. (2012) 28:974–8. doi: 10.3109/09513590.2012.683082, PMID: 22553983

[15] Pratama G. Mechanism of elevated LH/FSH ratio in lean PCOS revisited: a path analysis. Sci Rep. (2024) 14:8229. doi: 10.1038/s41598-024-58064-010.1038/s41598-024-58064-0, PMID: 38589425 PMC11002031

[16] Akpang N Kwiatkowski J Zaborowska L Ludwin A. Autoantibodies targeting the hypothalamic-pituitary-ovarian axis in polycystic ovary syndrome: emerging key players in pathogenesis? Int J Mol Sci. (2025) 26. doi: 10.3390/ijms26094121, PMID: 40362363 PMC12072038

[17] Norman RJ Dewailly D Legro RS Hickey TE. Polycystic ovary syndrome. Lancet. (2007) 370:685–97. doi: 10.1016/s0140-6736(07)61345-2, PMID: 17720020

[18] Sahmay S Atakul N Aydogan B Aydın Y Imamoglu M Seyisoglu H. Elevated serum levels of anti-Müllerian hormone can be introduced as a new diagnostic marker for polycystic ovary syndrome. Acta Obstetricia Gynecologica Scandinavica. (2013) 92:1369–74. doi: 10.1111/aogs.12247, PMID: 23980726

[19] Fauser BCJM Tarlatzis BC Rebar RW Legro RS Balen AH Lobo R. Consensus on women’s health aspects of polycystic ovary syndrome (PCOS): the Amsterdam ESHRE/ASRM-Sponsored 3rd PCOS Consensus Workshop Group. Fertility Sterility. (2012) 97:28–38.e25. doi: 10.1016/j.fertnstert.2011.09.024, PMID: 22153789

[20] Goyal M Dawood AS. Debates regarding lean patients with polycystic ovary syndrome: A narrative review. J Hum Reprod Sci. (2017) 10:154–61. doi: 10.4103/jhrs.JHRS_77_17, PMID: 29142442 PMC5672719

[21] Deswal R Yadav A Dang AS. Sex hormone binding globulin - an important biomarker for predicting PCOS risk: A systematic review and meta-analysis. Syst Biol Reprod Med. (2017) 64:12–24. doi: 10.1080/19396368.2017.1410591, PMID: 29227165

[22] Wang Y Gao H Di W Gu Z. Endocrinological and metabolic characteristics in patients who are non-obese and have polycystic ovary syndrome and different types of a family history of type 2 diabetes mellitus. J Int Med Res. (2021) 49. doi: 10.1177/03000605211016672, PMID: 34024175 PMC8142526

[23] Azziz R Woods KS Reyna R Key TJ Knochenhauer ES Yildiz BO. The prevalence and features of the polycystic ovary syndrome in an unselected population. J Clin Endocrinol Metab. (2004) 89:2745–9. doi: 10.1210/jc.2003-032046, PMID: 15181052

[24] Panidis D Tziomalos K Papadakis E Chatzis P Kandaraki EA Tsourdi EA. The clinical significance and primary determinants of hirsutism in patients with polycystic ovary syndrome. Eur J Endocrinol. (2013) 168:871–7. doi: 10.1530/eje-13-0039, PMID: 23557988

[25] Kim JJ. Obesity and polycystic ovary syndrome. J Obes Metab Syndrome. (2024) 33:289–301. doi: 10.7570/jomes24035, PMID: 39701598 PMC11704221

[26] Balen AH Laven JSE Tan SL Dewailly D. Ultrasound assessment of the polycystic ovary: international consensus definitions. Hum Reprod Update. (2003) 9:505–14. doi: 10.1093/humupd/dmg044, PMID: 14714587

[27] Kumar N Agarwal H. Early Clinical, Biochemical and radiological features in obese and non-obese young women with polycystic ovarian syndrome: A comparative study. Hormone Metab Res. (2022) 54:620–4. doi: 10.1055/a-1880-1264, PMID: 35858631

[28] Carmina E Lobo RA. Comparing lean and obese PCOS in different PCOS phenotypes: evidence that the body weight is more important than the rotterdam phenotype in influencing the metabolic status. Diagnostics. (2022) 12. doi: 10.3390/diagnostics12102313, PMID: 36292002 PMC9600591

[29] Arunachalam R Priya K Brindha R Parthiban K. Understanding metabolic patterns in polycystic ovary syndrome: Comparing lean and obese women at a family medicine clinic. J Family Med Primary Care. (2024) 13:1837–42. doi: 10.4103/jfmpc.jfmpc_1425_23, PMID: 38948599 PMC11213445

[30] Al-Ruthia YS Al-Mandeel H AlSanawi H Mansy W AlGasem R AlMutairi L. Ovulation induction by metformin among obese versus non-obese women with polycystic ovary syndrome. Saudi Pharm J. (2017) 25:795–800. doi: 10.1016/j.jsps.2016.12.001, PMID: 28725153 PMC5506743

