# Supplementary material for: Analyzing and evaluating the metabolic and endocrine characteristics between lean and obese patients with polycystic ovary syndrome: a systemic review and meta-analysis
Source: Front Endocrinol (Lausanne). 2025 Oct 14;16:1680685. doi: 10.3389/fendo.2025.1680685 (PMC12558778; doi:10.3389/fendo.2025.1680685)
Supplement: Supplementary file 1 [file Table1.docx]

**Supplementary appendix**

**Contents**

[**Supplement Table 1. Full search strategy. 1**](#_Toc197630718)

[**Supplement Table 2. Characteristics of studies and patients’ baseline characteristics. 3**](#_Toc197630719)

[**Supplement Table 3. Quality judgments about each risk of bias item. 8**](#_Toc197630720)

# Supplement Table 1. Full search strategy.

**PubMed**

| # | Searches | Results |
| --- | --- | --- |
| 1 | ((((((((((polycystic ovary syndrome [MeSH Terms]) | **19,873** |
| 2 | (polycystic ovary syndrome[Title/Abstract])) OR (polycystic ovar*[Title/Abstract])) OR (poly-cystic ovar*[Title/Abstract])) OR ((PCOS[Title/Abstract] ))) OR ((Stein-Leventhal[Title/Abstract] OR Leventhal[Title/Abstract]))) OR (anovulation[Title/Abstract])) OR (anovulat*[Title/Abstract])) OR (oligo-ovulat*[Title/Abstract])) OR (oligoovulat*[Title/Abstract])) OR (sclerocystic ovary syndrome[Title/Abstract]) | **30,768** |
| 3 | #1 OR #2 | **32,272** |
| 4 | ((((thinness [MeSH Terms]) OR (underweight [MeSH Terms])) OR (obesity [MeSH Terms])) OR (overweight [MeSH Terms])) | **305,448** |
| 5 | ((((((((((((((((((((obes*[Title/Abstract])) OR (body mass ind*[Title/Abstract])) OR (adipos*[Title/Abstract])) OR (overweight[Title/Abstract])) OR (overload syndrom*[Title/Abstract])) OR (overeat*[Title/Abstract])) OR (over eat*[Title/Abstract])) OR (overfeed*[Title/Abstract])) OR (over feed*[Title/Abstract])) OR (overfed[Title/Abstract])) OR (over fed[Title/Abstract])) OR (weight cycling[Title/Abstract])) OR (skinfold thickness[Title/Abstract])) OR (antiobesity[Title/Abstract])) OR (anti-obesity[Title/Abstract])) OR (obesitas[Title/Abstract])) OR (bodyweight[Title/Abstract])) OR (body weight[Title/Abstract])) OR ((((( (underweight[Title/Abstract])) OR (normal weight[Title/Abstract])) OR (normal weight[Title/Abstract])) OR (nonoverweight[Title/Abstract])) OR (non obesity[Title/Abstract])) OR (nonobesity[Title/Abstract])) OR (underweight[Title/Abstract]))) OR ((((((thinness[Title/Abstract])) OR (thin[Title/Abstract])) OR (thinness[Title/Abstract])) OR (lean[Title/Abstract])) OR (non obese[Title/Abstract])) OR (nonobese[Title/Abstract])) | **1,168,343** |
| 6 | #4 OR #5 | **1,226,601** |
| 7 | #3 AND #6 | **10,518** |

**ISI Web of Science**

| **#** | Searches | Results |
| --- | --- | --- |
| 1 | ((((((((((TS=(polycystic ovary syndrome)) OR TS=(polycystic ovar*)) OR TS=(poly-cystic ovar*)) OR TS=(PCOS)) OR TS=(Stein-Leventhal)) OR TS=(Leventhal)) OR TS=(anovulation)) OR TS=(anovulat*)) OR TS=(oligo-ovulat*)) OR TS=(oligoovulat*)) OR TS=(sclerocystic ovary syndrome) | **53,429** |
| 2 | ((((((((((((((((((((((((((((((((TS=(thinness)) OR TS=(thin)) OR TS=(thinness)) OR TS=(lean)) OR TS=(non obese)) OR TS=(nonobese)) OR TS=(underweight)) OR TS=(normal weight)) OR TS=(normal weight)) OR TS=(nonoverweight)) OR TS=(non obesity)) OR TS=(nonobesity)) OR TS=(underweight)) OR TS=(obesity)) OR TS=(overweight)) OR TS=(weight loss)) OR TS=(obes*)) OR TS=(body mass ind*)) OR TS=(adipos*)) OR TS=(overload syndrom*)) OR TS=(overeat*)) OR TS=(over eat*)) OR TS=(overfeed*)) OR TS=(over feed*)) OR TS=(overfed)) OR TS=(over fed)) OR TS=(weight cycling)) OR TS=(skinfold thickness)) OR TS=(antiobesity)) OR TS=(anti-obesity)) OR TS=(obesitas)) OR TS=(bodyweight)) OR TS=(body weight) | **8,290,375** |
| 3 | #1 AND #2 | **20,885** |

# Supplement Table 2. Characteristics of studies and patients’ baseline characteristics.

| Study and year | Location | Sample size | Number of lean (L) and obese (O) PCOS | Age, lean (L) and obese (O) PCOS, mean (SD), mean (min-max), mean (SE), median (5-95% CI), mean (5-95% CI), or medians (interquartile ranges), y | BMI, lean (L) and obese (O) PCOS, mean (SD), mean (min-max), mean (SE), median (5-95% CI), mean (5-95% CI), or medians (interquartile ranges), kg/m^2^ |
| --- | --- | --- | --- | --- | --- |
| Abdul-Maksoud2020 | Egypt | 140 | L-70; O-70 | L-34.4 (4.8)  O-34.7 (4.4) | L-20.5 (1.6)  O-31.6 (2.5) |
| Adamska2013 | Poland | 92 | L-40; O-52 | L-23.9 (4.1)  O-26.4 (6.6) | L-21.4 (2.0)  O-31.2 (4.1) |
| Agacayak2015 | Turkey | 30 | L-15; O-15 | L-24.4 (4.0)  O-28.0 (4.0) | L-20.3 (2.2)  O-27.3 (2.0) |
| Agar2022 | Turkey | 50 | L-25; O-25 | NA | NA |
| Alatas2020 | Turkey | 57 | L-28; O-29 | L-22.6 (5.0)  O-27.2 (5.7) | L-20.6 (1.3)  O-28.4 (3.0) |
| Anttila1991 | Finland | 24 | L-14; O-10 | L-34.4 (4.8)  O-34.7 (4.4) | L-21.0 (1.6)  O-31.7 (4.6) |
| Arunachalam2024 | India | 51 | L-13; O-38 | NA | NA |
| Bahceci2021 | Turkey | 60 | L-30; O-30 | L-22.5 (18-30) ^*^  O-22 (18-33) ^*^ | L-21.9 (15.8-24.9) ^*^  O-29.2 (25.1-37.2) ^*^ |
| Bernasconi1996 | Italy | 112 | L-60; O-52 | L-22.5 (5.3)  O-23.2 (6.9) | L-20.9 (1.9)  O-31.5 (3.6) |
| Bousmpoula2017 | Greece | 60 | L-30; O-30 | L-32.1 (4.2)  O-32.3 (3.9) | L-22.4 (1.0)  O-26.9 (1.5) |
| Chen2012 | China | 239 | L-135; O-104 | L-26.1 (3.0)  O-28.0 (4.2) | L-20.6 (2.3)  O-31.2 (4.7) |
| Chen2014 | China | 224 | L-103; O-121 | L-25.1 (5.7)  O-27.0 (5.8) | L-20.4 (1.7)  O-30.4 (3.6) |
| Cho2017 | Qatar | 25 | L-11; O-14 | L-23.3 (4.5)  O-26.0 (4.1) | L-22.9 (1.4)  O-36.8 (4.8) |
| Dadachanji2015 | India | 285 | L-108; O-177 | L-23.44 (4.13)  O-25.9 (5.24) | L-20.05 (1.98)  O-28.62 (4.43) |
| Dale1992 | Norway | 49 | L-19; O-30 | L-29.0 (0.8)  O-28.0 (0.7) | L-21.4 (0.5)  O-30.5 (0.7) |
| Durmus2016 | Turkey | 76 | L-38; O-38 | L-21.5 (18-30) ^*^  O-22.5 (18-38) ^*^ | L-22.5 (18.3-24.7) ^*^  O-31.4 (25.9-38.4) ^*^ |
| Economou2009 | Greece | 83 | L-44; O-39 | L-23.6 (4.5)  O-26.4 (4.8) | L-22.0 (1.6)  O-30.1 (2.8) |
| Er Luo2021 | China | 156 | L-50; O-106 | L-29.48 (2.94)  O-30.72 (3.1) | L-20.94 (1.55)  O-27.13 (2.77) |
| Erel2003 | Turkey | 44 | L-25; O-19 | L-22.0 (3.5)  O-26.5 (4.5) | L-21.5 (2.2)  O-31.8 (5.6) |
| Fahad2023 | Malaysia | 100 | L-30; O-70 | L-30.97 (5.64)  O-32.67 (6.00) | L-23.23 (1.65)  O-29.29 (3.05) |
| Faloia2004 | Italy | 50 | L-23; O-27 | L-23 (4.9)  O-21 (4.8) | L-22.0 (2.0)  O-32.6 (5.3) |
| Feng2019 | China | 71 | L-29; O-42 | L-30.72 (3.01)  O-31.43 (3.28) | L-21.19 (1.62)  O-26.36 (1.86) |
| Gao2023 | China | 78 | L-38; O-40 | L-24.52 (4.7)  O-25.26 (5.7) | L-19.92 (3.51)  O-31.40 (5.40) |
| Grulet1993 | France | 61 | L-30; O-31 | NA | L-21.75 (1.95)  O-33.30 (2634) |
| Guzel2014 | Turkey | 80 | L-40; O-40 | L-23.35 (5.49)  O-28.11 (5.63) | L-20.99 (2.37)  O-31.47 (5.08) |
| Inal2015 | Turkey | 60 | L-30; O-30 | L-29.40 (3.70)  O-29.97 (3.66) | NA |
| Ipsita2024 | India | 143 | L-35; O-108 | L-22.94 (5.677)  O-28.05 (5.722) | NA |
| Kahraman2019 | Turkey | 52 | L-21; O-31 | L-24.76 (3.88)  O-24.13 (4.84) | L-21.82 (2.24)  O-31.48 (4.47) |
| Kamardi2021 | Indonesia | 55 | L-38; O-17 | L-30.1 (4.0)  O-30.4 (4.3) | L-22.5 (1.3)  O-29.4 (3.4) |
| Kandaraki2011 | United  Kingdom | 71 | L-38; O-33 | L-29.32 (5.71)  O-27.15 (2.10) | L-21.37 (1.90)  O-34.55 (5.66) |
| Keskinkurt2014 | Turkey | 62 | L-30; O-32 | L-32.8 (5.0)  O-31.6 (3.0) | L-22.4 (2.5)  O-31.9 (4.1) |
| Keyif2020 | Turkey | 35 | L-17; O-18 | L-28.88 (4.21)  O-29.06 (2.86) | L-20.78 (2.23)  O-33.81 (4.36) |
| Kiddy1990 | United  Kingdom | 263 | L-172; O-91 | NA | L-21.0 (1.8)  O-30.3 (4.2) |
| Kikuchi2002 | Japan | 40 | L-20; O-20 | L-30.8 (1.37) ^†^  O-31.7 (1.22) ^†^ | L-20.83 (0.43) ^†^  O-29.05 (0.68) ^†^ |

| Kowalska2007 | Poland | 71 | L-23; O-47 | L-23.69 (3.46)  O-26.13 (6.19) | L-21.39 (2.10)  O-30.99 (3.99) |
| --- | --- | --- | --- | --- | --- |
| Layegh2016 | Iran | 115 | L-45; O-70 | L-22.53 (4.46)  O-25.84 (5.67) | NA |
| Lee2012 | Korea | 144 | L-69; O-75 | L-25 (4)  O-27 (5) | L-20.6 (1.6)  O-27.2 (2.7) |
| Lee2013 | Korea | 40 | L-20; O-20 | L-24 (5)  O-26 (7) | L-21.9 (2.0)  O-27.4 (1.9) |
| Li2016 | China | 32 | L-11; O-21 | L-25.7 (4.4)  O-29.3 (6.5) | L-21.7 (2.2)  O-30.0 (3.6) |
| Li2024 | China | 255 | L-145; O-110 | L-24.22 (4.61)  O-25.06 (4.77) | NA |
| Lin2015 | China | 30 | L-15; O-15 | L-25.3 (1.60) ^†^  O-28.0 (1.48) ^†^ | L-21.5 (0.54) ^†^  O-28.7 (0.82) ^†^ |
| Liou2009 | China | 295 | L-180; O-115 | L-26.2 (5.2)  O-27.6 (5.5) | L-20.3 (2.1)  O-31.2 (4.4) |
| Liu2022 | China | 112 | L-44; O-68 | L-26.56 (4.23)  O-25.73 (5.20) | L-22.82 (1.12)  O-28.84 (2.08) |
| Makhija2023 | India | 96 | L-30; O-66 | NA | NA |
| Mancini2009 | Italy | 24 | L-14; O-10 | NA | NA |
| Misra2024 | India | 80 | L-28; O-52 | L-24.53 (3.58)  O-26.31 (4.36) | L-20.42 (1.86)  O-28.74 (4.21) |
| Morciano2014 | Italy | 399 | L-201; O-198 | L-29 (4)  O-28 (4) | L-22.81 (19-24.53) ^*^  O-28.42 (26-31) ^*^ |
| Naina2022 | India | 80 | L-40; O-40 | L-20.25 (1.45)  O-20.43 (1.53) | L-22.58 (2.10)  O-28.36 (2.47) |
| Nayak2020 | India | 287 | L-112; O-175 | L-21.39 (4.23)  O-23.13 (6.10) | L-20.18 (1.87)  O-27.93 (5.26) |
| Nikolajuk2010 | Poland | 78 | L-35; O-43 | L-24.11 (3.94)  O-25.60 (5.57) | L-21.71 (1.81)  O-31.46 (4.34) |
| Pande2017 | India | 124 | L-53; O-71 | L-22 (15-28.3) ^‡^  O-23 (15.6-34.4) ^‡^ | L-20.7  (16.26-22.83) ^‡^  O-28.62  (23.4-37.94) ^‡^ |
| Pangaribuan2011 | Indonesia | 24 | L-10; O-14 | L-25.6 (6.1)  O-28.1 (4.7) | L-22.0 (1.7)  O-28.6 (3.1) |
| Park2015 | Korea | 458 | L-352; O-106 | L-26.2 (3.8)  O-28.2 (4.7) | L-20.4 (2.0)  O-28.2 (3.6) |
| Patlolla2017 | India | 52 | L-15; O-37 | L-21.37 (3.2)  O-25.69 (5.9) | NA |
| Rajkhowa1995 | Finland | 153 | L-38; O-115 | NA | NA |
| Saadia2020 | Qatar | 63 | L-30; O-33 | L-24.8 (5.1)  O-25.6 (7.4) | L-21.5 (1.7)  O-30.3 (4.3) |
| Satyaraddi2019 | India | 81 | L-39; O-42 | L-25.4 (3.8)  O-25.2 (3.9) | L-22.2 (2.4)  O-30.9 (4.9) |
| Shabir2013 | India | 197 | L-97; O-100 | L-22.90 (5.7)  O-24.17 (6.2) | L-21.4 (2.2)  O-29.4 (3.5) |
| Sharifi2010 | Iran | 103 | L-34; O-69 | NA | NA |
| Shi2020 | China | 80 | L-32; O-48 | L-30.31 (2.93)  O-31.15 (3.68) | L-21.21 (1.55)  O-26.55 (1.86) |
| Svendsen2008 | Denmark | 35 | L-17; O-18 | L-28 (4.7)  O-29 (3.9) | L-23 (1.5)  O-33 (4.0) |
| Svendsen2009 | Denmark | 36 | L-17; O-19 | L-28(5)  O-29 (4) | L-23 (2)  O-33 (4) |
| Tao2012 | China | 147 | L-59; O-78 | L-25.4 (24.1-26.8) ^§^  O-26.6 (24.8-28.4) ^§^ | L-20.8 (20.3-21.4) ^§^  O-29.8 (28.9-30.6) ^§^ |
| Usta2018 | Turkey | 61 | L-31; O-30 | L-24.7 (4.9)  O-24.6 (4.6) | L-22.3 (3.7)  O-29.1 (4.2) |
| Vasyukova2023 | Russia | 44 | L-19; O-25 | L-27 (24-30) ^¶^  O-27 (22.5-30.5) ^¶^ | L-21.9 (20-23.8) ^¶^  O-33.9 (28.2-37.9) ^¶^ |
| Villa1999 | Italy | 22 | L-12; O-10 | NA | L-21.9 (1.5)  O-34.0 (4.5) |
| Wang2010 | China | 46 | L-20; O-26 | NA | NA |
| Wang2018 | China | 61 | L-30; O-31 | L-24.5 (21-31.25) ^¶^  O-26 (23-31) ^¶^ | L-21.7  (19.41-23.3) ^¶^  O-26.89  (25.63-27.77) ^¶^ |
| Yang2015 | China | 118 | L-58; O-60 | L-24.61 (4.41)  O-25.50 (4.12) | L-20.86 (2.16)  O-28.28 (2.41) |
| Yildizhan2008 | Turkey | 67 | L-39; O-28 | L-25.81 (3.07)  O-28.46 (4.42) | L-22.26 (1.89) |
|  |  |  |  |  | O-29.74 (2.59) |
| Yildizhan2009 | Turkey | 100 | L-43; O-57 | L-26.67 (3.62)  O-25.51 (3.91) | L-22.17 (1.86)  O-32.84 (5.43) |
| Yilmaz2005 | Turkey | 85 | L-47; O-38 | L-22.95 (5.49)  O-23.51 (6.18) | L-20.99 (2.14)  O-31.55 (5.78) |
| Yilmaz2015 | Turkey | 41 | L-16; O-25 | L-21.81 (3.97)  O-25.12 (4.76) | L-21.79 (2.23)  O-33.62 (5.48) |

^*^: mean (min-max)

^†^: mean (SE)

^‡^: median (5–95 CI)

^§^: mean (5–95 CI)

^¶^: medians (interquartile ranges)

# Supplement Table 3. Quality judgments about each risk of bias item.

| Study | Selection | Comparability | Outcome |
| --- | --- | --- | --- |
| Abdul-Maksoud2020 | ******* | ***** | ******* |
| Adamska2013 | ****** | **-** | ******* |
| Agacayak2015 | ******* | **-** | ***** |
| Agar2022 | ****** | **-** | ***** |
| Alatas2020 | ******* | ***** | ****** |
| Anttila1991 | ****** | ***** | ***** |
| Arunachalam2024 | ******* | ***** | ******* |
| Bahceci2021 | ****** | ***** | ******* |
| Bernasconi1996 | ******* | ***** | ****** |
| Bousmpoula2017 | ****** | ***** | ******* |
| Chen2012 | ******* | ***** | ****** |
| Chen2014 | ******* | ***** | ****** |
| Cho2017 | ****** | ***** | ******* |
| Dadachanji2015 | ******** | ***** | ******* |
| Dale1992 | ******* | ***** | ****** |
| Durmus2016 | ******** | ***** | ****** |
| Economou2009 | ******* | ***** | ******* |
| Er Luo2021 | ******** | ***** | ****** |
| Erel2003 | ******* | ***** | ******* |
| Fahad2023 | ******** | ***** | ****** |
| Faloia2004 | ******* | ***** | ******* |
| Feng2019 | ******** | ***** | ****** |
| Gao2023 | ******* | ***** | ******* |
| Grulet1993 | ******* | ***** | ******* |
| Guzel2014 | ****** | **-** | ****** |
| Inal2015 | ******* | ***** | ******* |
| Ipsita2024 | ****** | ***** | ****** |
| Kahraman2019 | ******* | ***** | ****** |
| Kamardi2021 | ******* | ***** | ******* |
| Kandaraki2011 | ****** | ***** | ******* |
| Keskinkurt2014 | ******** | ***** | ****** |
| Keyif2020 | ******* | **-** | ****** |
| Kiddy1990 | ******** | ***** | ******* |
| Kikuchi2002 | ******* | ***** | ****** |
| Kowalska2007 | ******** | ***** | ******* |
| Layegh2016 | ******* | ***** | ****** |
| Lee2012 | ******* | ***** | ******* |
| Lee2013 | ****** | ***** | ****** |
| Li2016 | ******* | **-** | ******* |
| Li2024 | ****** | ***** | ****** |
| Lin2015 | ******* | ***** | ******* |
| Liou2009 | ******* | ***** | ****** |
| Liu2022 | ****** | ***** | ****** |
| Makhija2023 | ******** | ***** | ******* |
| Mancini2009 | ******* | ***** | ******* |
| Misra2024 | ******** | **-** | ****** |
| Morciano2014 | ******* | ***** | ****** |
| Naina2022 | ******** | ***** | ******* |
| Nayak2020 | ******* | ***** | ****** |
| Nikolajuk2010 | ******* | ***** | ******* |
| Pande2017 | ****** | ***** | ****** |
| Pangaribuan2011 | ******* | ***** | ******* |
| Park2015 | ****** | **-** | ****** |
| Patlolla2017 | ******* | ***** | ******* |
| Rajkhowa1995 | ******* | ***** | ****** |
| Saadia2020 | ****** | ***** | ******* |
| Satyaraddi2019 | ******** | ***** | ****** |
| Shabir2013 | ******* | ***** | ****** |
| Sharifi2010 | ******** | ***** | ******* |
| Shi2020 | ******* | **-** | ******* |
| Svendsen2008 | ******** | ***** | ****** |
| Svendsen2009 | ******* | ***** | ****** |
| Tao2012 | ******* | ***** | ******* |
| Usta2018 | ****** | ***** | ****** |
| Vasyukova2023 | ******* | ***** | ******* |
| Villa1999 | ****** | ***** | ****** |
| Wang2010 | ******* | **-** | ******* |
| Wang2018 | ******* | ***** | ****** |
| Yang2015 | ****** | ***** | ******* |
| Yildizhan2008 | ******** | ***** | ****** |
| Yildizhan2009 | ******* | ***** | ******* |
| Yilmaz2005 | ******** | ***** | ****** |
| Yilmaz2015 | ******* | ***** | ******* |

^*^: 1 score

^**^: 2 score

^***^: 3 score

^****^: 4 score
